# Executing cell-specific cross-linking immunoprecipitation and sequencing (seCLIP) in *C. elegans*

**DOI:** 10.1016/j.xpro.2022.101959

**Published:** 2022-12-24

**Authors:** Stephen M. Blazie, Yishi Jin

**Affiliations:** 1 Department of Neurobiology, School of Biological Sciences, University of California, San Diego, La Jolla, CA 92093, USA

**Keywords:** Genetics, Genomics

## Abstract

The single-end enhanced cross-linking immunoprecipitation (seCLIP) method is well suited for efficient and unbiased transcriptome-wide interrogation of RNA-binding protein (RBP) interaction sites. Here, we provide a protocol for executing cell-specific seCLIP for any desired RBP in *Caenorhabditis elegans*. We begin with steps and recommendations for transgene construction and Cas9-mediated chromosomal integration. We provide detailed procedures for isolation of RBP-associated RNA fragments, subsequent library preparation, and sequencing. We further discuss best practices for data analysis, interpretation of results, and troubleshooting.

For complete details on the use and execution of this protocol, please refer to Blazie et al. (2021).^1^

## Highlights

• A step-by-step cell-specific seCLIP-seq protocol in *C. elegans*
• Guidelines for transgene design and Cas9-mediated single-copy chromosomal insertion
• Advice for computational mapping of RBP interaction sites across the transcriptome

## Abstract (editor highlights)

Publisher’s note: Undertaking any experimental protocol requires adherence to local institutional guidelines for laboratory safety and ethics.

## Graphical abstract

**Figure:**
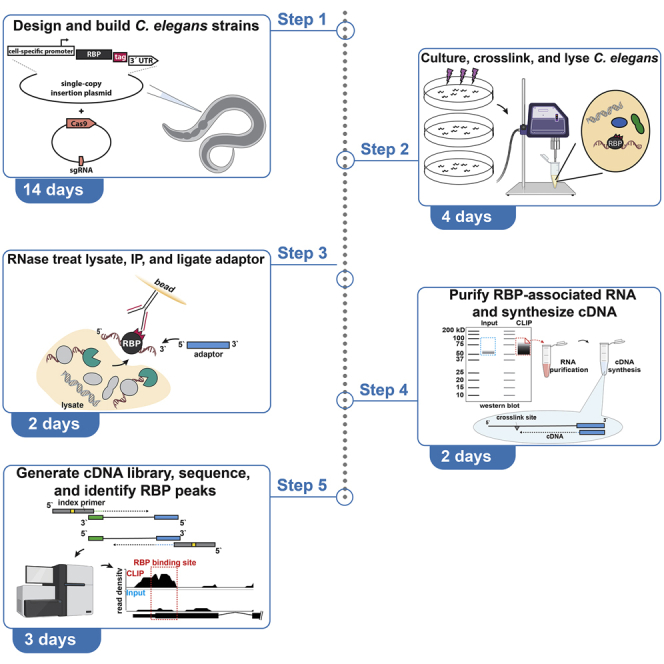

## Before you begin

The goal of this method is to identify transcriptome-wide interaction sites for an RBP in specific cells in *C. elegans*. This method relies on expressing an epitope-tagged RBP transgene under a cell-specific promoter. In the following section, we outline recommended steps and considerations for designing RBP transgene expressing strains in preparation for seCLIP.

### seCLIP controls

RBP binding sites are identified from seCLIP sequencing data as clusters of aligned sequencing reads.^2^ However, many read clusters derive from non-specific association with common RNA ‘contaminants’ during immunoprecipitation, especially short, abundant transcripts (e.g., *trans*-splice leader transcripts and small nucleolar RNAs). Therefore, it is important to implement controls to help distinguish signal from noise in seCLIP sequencing data. At minimum, we recommend simultaneously performing seCLIP with a negative control strain lacking any RBP transgene (e.g., N2 strain), which is useful for identifying non-specific transcripts that stick to beads during immunoprecipitation (IP) (Blazie et al.^1^). Sequenced read clusters identified from this ‘no transgene’ control can be regarded as background and subtracted from the RBP transgene data sets. We also recommend including a control transgene that expresses a mutated or truncated RBP lacking RNA binding activity. This ‘inactive RBP’ control is especially useful when the experimenter desires to pinpoint RNA binding sites for a specific RBP (target RBP) that co-immunoprecipitates with other RBPs (off-target RBPs). Performing seCLIP with the ‘inactive RBP’ control is crucial for identifying the off-target RBP-binding sites, which can be filtered from sample datasets to refine a list of high-confidence binding sites of the target RBP (see data analysis for more details).

In summary, we recommend including two negative control strains to aid interpretation of seCLIP sequencing data: 1) a strain lacking the transgene (wild type), and 2) a strain expressing an inactive RBP transgene.

### Transgene design

Cell-specific seCLIP involves generating strains expressing an epitope-tagged RBP transgene with a cell-specific promoter. Consider the following aspects of transgene design before you begin:

Promoter: The choice of promoter will depend on which cells are intended for profiling RBP activity. The ideal promoter will drive transgene expression in the correct cells and within the desired temporal context, with limited background in other cells. Beware of promoters that greatly over-express the RBP as they could induce cell toxicity or lead to dosage-related RBP-binding artifacts.^3^ Numerous *C. elegans* cell-specific promoters have been well characterized and exploited for cell-specific transcriptome studies.^4^^,^^5^^,^^6^^,^^7^

Epitope tag: There are many epitope tags well-suited for seCLIP in *C. elegans* (e.g., FLAG, HA, Myc, V5, GFP). As the epitope tag will determine which reagents are needed for the immunoprecipitation (IP) step of seCLIP, it is important to evaluate whether IP efficiency is suitable with a given tag. Parameters influencing RBP activity should also be considered, such as tag size, amino acid composition, and its placement on the N- or C-terminus of the RBP. Standard fluorescent proteins (GFP and RFP) often serve as effective epitope tags in cases where fluorescence is desired to visualize protein expression. However, the large size of fluorescent proteins could alter the activity of some RBPs. We generally recommend using a short tag such as 3×FLAG or HA as their small size is less likely to interfere with RBP function. The impact of other epitope tag parameters (C or N-terminal placement and composition) heavily depends on the nature of the RBP being tagged. In summary, it is critical to verify that the tagged RBP retains function using transgene rescue or other strategies.

### Choice of transgenesis method

Choosing the best transgenesis method is essential to obtain biologically meaningful results. Each *C. elegans* transgenesis method has advantages and disadvantages. We recommend generating single-copy transgene insertion strains as they have the advantage of expressing the transgene in all intended cells (no mosaicism) and avoiding over-expression associated with multi-copy extrachromosomal arrays. Although several methods for single-copy insertion have been developed over the years, here we will describe steps used to generating strains using the Cas9-mediated single copy insertion method (CasSCI).

### CasSCI transgenesis

**Timing: ∼2 weeks**

**Timing: 4–5 days (for step 1)**

**Timing: 4 days (for step 2)**

**Timing: 5 days (for step 3)**

**Figure 1:**
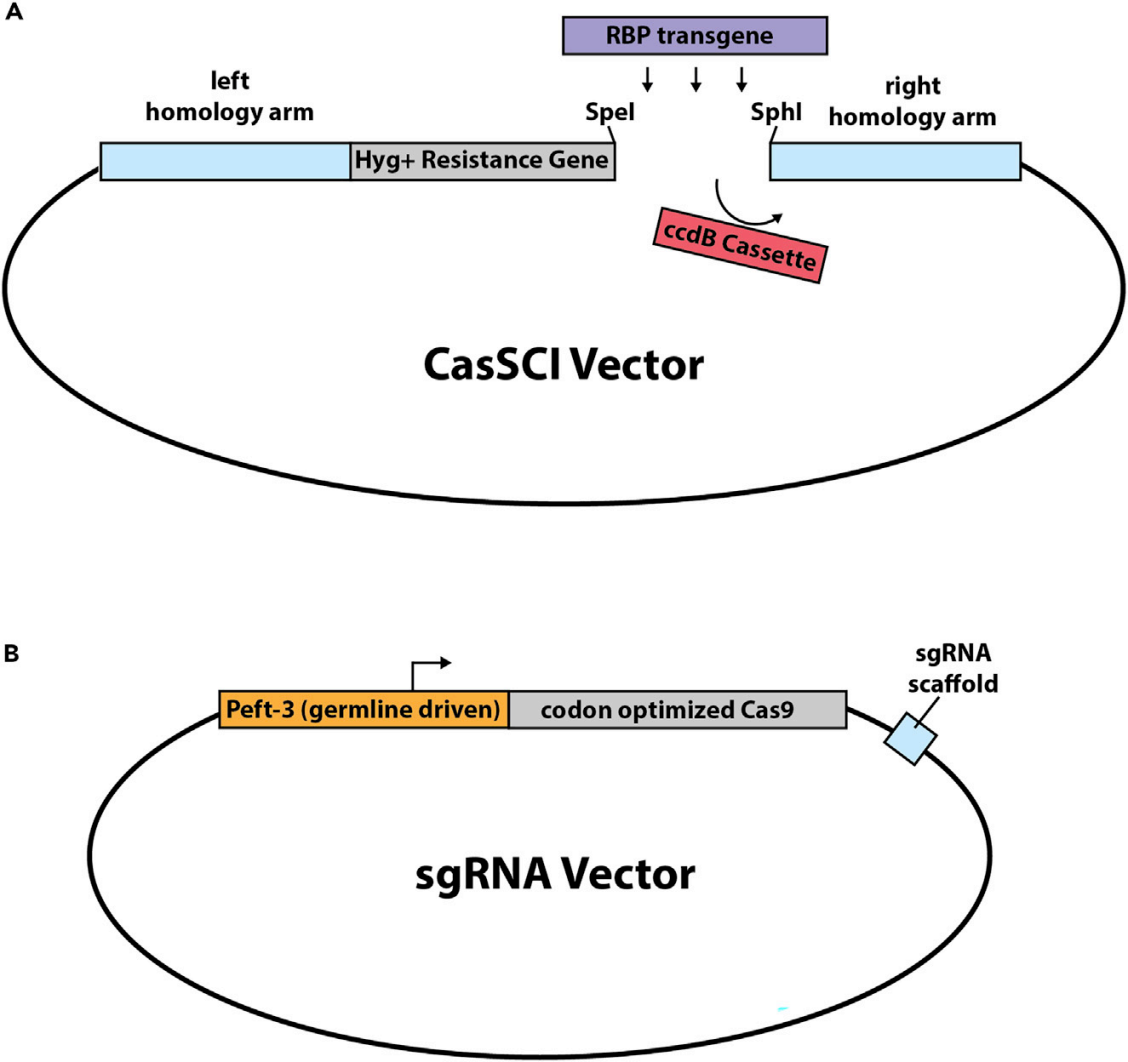
Architecture of the CasSCI and sgRNA vectors (A) CasSCI vectors contain a hygromycin (Hyg+) resistance gene and ccdB cassette flanked by sequences with homology to the chromosomal insertion site (∼1,500 bp each). SpeI and SphI restriction enzyme sites facilitate RBP transgene cloning in place of the ccdB cassette. (B) sgRNA vectors contain a *C. elegans* codon optimized Cas9 gene driven by the *Peft-3* germline specific promoter and an sgRNA scaffold designed for the chromosomal insertion site. Note that illustrations in A and B are not to scale.

CasSCI is a CRISPR-based approach to knock-in transgenes to intergenic euchromatic loci,^8^ inspired by the Mos1 transposon single copy insertion (MosSCI) technology.^9^ In CasSCI, the transgene is first cloned between sequences with homology to the chosen chromosomal locus (Figure 1A). The CasSCI vector also contains a positive-selection Hygromycin resistance cassette, which is located next to the transgene such that both are inserted into the chromosome (Figure 1A). The CasSCI vector is then co-microinjected with a plasmid encoding an sgRNA and Cas9 (derived from pDD162,^10^), which facilitate CRISPR germline insertion of the transgene in the germline (Figure 1B). Fluorescent markers expressed in the pharynx (*Pmyo-2*::mCherry) and muscle (*Pmyo-3*::mCherry) are also co-microinjected and serve to 1) identify successful microinjection events indicated by fluorescent F1 progeny, and 2) distinguish extrachromosomal array transgenics from single-copy insertion animals. The F1 progeny of the injected P0 animals are then treated with hygromycin and in several days single-copy insertion animals are identified on the basis of 1) resistance to hygromycin, and 2) lacking expression of the co-injection markers. Correctly integrated single-copy insertions can then be verified using three primers in a single PCR reaction. Two of the genotyping primers bridge the chromosomal insertion site (e.g., YJ10507/YJ10508 for Chr I) and yield a product from wild type alleles where no transgene insertion has ocurred. The third primer (YJ10686) anneals within the inserted hygromycin cassette and works with the chromosome specific primer (e.g., YJ10507 for Chr I) to yield a product from insertion alleles. Importantly, including all three primers in the PCR reaction will produce two different size products if both wild type and insertion alleles are present, allowing the user to distinguish between heterozygous and homozygous insertion animals.

In the following section, we describe the steps needed to generate a CasSCI transgenic line.

1. Clone the cell-specific transgene encoding epitope-tagged RBP into CasSCI vector for single-copy genomic insertion.
  ***Note:*** We use Gibson Assembly technology^11^ to clone the RBP transgene into the CasSCI vector. The choice of CasSCI vector (available in AddGene) will depend on the intended insertion site: use pCZGY2727 (for insertions on Chromosome I; *ttTi4348*) or pCZGY2729 (Chr IV; *cxTi10882*). Transgene cloning is facilitated by using SpeI and SphI restriction enzyme sites present on each CasSCI vector. See troubleshooting, problem 1 for advice on purifying the CasSCI vector.
  a. Design oligonucleotides for transgene assembly (see Note below).
    **CRITICAL:** Aim for at least 20 nucleotides and annealing temperature of ∼60°C for the 3′ region of the primer annealing to the target sequence.
    ***Note:*** We typically PCR amplify three DNA fragments corresponding to the promoter sequence, the epitope tag, and the RBP cDNA (including 3′ UTR) with a high-fidelity (e.g., Phusion) polymerase. Design primers with 3′ ends annealing to the target sequence. The 5′ end of the primers should contain 20 additional nucleotides, which do not anneal to the target, but are instead homologous to the adjacent DNA fragment to facilitate Gibson assembly. For example, the 5′ end of the forward primer used to amplify the promoter will contain 20 nucleotides homologous to the CasSCI vector sequence and the promoter reverse primer will contain 20 nucleotides homologous to the epitope tag-RBP. NEBbuilder is a useful tool for designing Gibson Assembly primers and checking plasmids designed with Gibson Assembly.
  b. Digest the CasSCI vector (e.g., pCZGY2727) with SpeI and SphI (37°C for 1 h, then deactivate at 80°C for 20 min).
  c. Check products on a 1% standard agarose gel to ensure correct digestion (2 bands: ∼10.5 kb vector backbone and 1.8 kb insert).
  d. Prepare the Gibson assembly reaction (10 μL total volume):
    i. Add 1 μL each PCR product and 1 μL digested CasSCI vector to a PCR tube, then add ddH_2_O to 5 μL.
    ii. Add 5 μL Gibson Assembly Master Mix to the reaction.
    iii. Flick tube to mix, then briefly spin down.
      ***Note:*** Gel extraction of the digested CasSCI vector backbone is not necessary as long as the SpeI and SphI enzymes were deactivated as instructed in step b.
      **CRITICAL:** We highly recommend setting up a control reaction with the Gibson assembly control DNA mix: 5 μL Gibson Assembly Control + 5 μL Gibson Assembly Master Mix. This can be used to verify the cloning reaction is working.
  e. Incubate the reaction(s) at 50°C for 1 h.
  f. Transform 1 μL of the reaction into DH5-alpha competent cells and spread on Ampicillin LB plates.
  g. Screen several (typically 4) clones to verify correct assembly using restriction digest.
    **CRITICAL:** We further recommend sequencing the final clone to verify mutations were not introduced during PCR.

2. Generation of *C. elegans* strains expressing cell-specific RBP transgenes.
  a. Determine which sgRNA vector is required for the chosen insertion site: pCZGY2748 (Chr I; ttTi4348) or pCZGY2750 (Chr IV; cxTi10882).
  b. Prepare CasSCI microinjection mix: 5–25 ng/μL RBP transgene in CasSCI vector (see Note below), 15 ng/μL sgRNA vector, 2 ng/μL pCFJ90 (*Pmyo-2*::mCherry), 5 ng/μL pCFJ104 (*Pmyo-3*::mCherry), 30 ng/μL DNA ladder.
    ***Note:*** The amount of transgene to inject depends on the toxicity level of the RBP gene and should be determined empirically by titrating transgene concentration in microinjections until evidence of transgenesis (co-injection marker expression) is observed. The presence of sick or arrested F1 transgenic larval animals is a sign of transgene toxicity. As a guideline, we routinely obtained single-copy *eif-3.G* transgene insertion lines when injecting the transgene at 5 ng/μL, but very few transgene insertions when injecting more than 10 ng/μL.
  c. Microinject the DNA mixture into ∼30–40 P0 young adult hermaphrodite N2 animals following standard procedure (Mello and Fire, 1995).
  d. ∼1 h after microinjection, move the P0 animals to fresh 10 mm NGM plates seeded with OP50 (1–2 animals per plate) and culture at 25°C for 3 days.
3. Selection of transgenic animals.
  a. After the incubation period, observe the plates under a fluorescent dissection microscope for evidence that the microinjections were successful.
    ***Note:*** For 1–2 P0 animals plated, a good injection event will typically contain 15–20 fluorescent F1s. This step is merely to check that microinjection has worked. However, we recommend treating all plates (whether or not they contain fluorescent animals) with hygromycin as they may contain successful insertion events.
  b. Prepare hygromycin solution (300 μL for each plate) by diluting 60 μL of the hygromycin stock (50 mg/mL) into 240 μL ddH_2_O (for 60 mm NGM plates; adjust accordingly if using larger plates).
  c. Add 300 μL hygromycin solution to each NGM plate with fluorescent animals.
    i. Gently swirl the solution around the plates so it covers the plate surface.
    ii. Leave the lids off the plates to dry for ∼15 min at room temperature (20°C–23°C).
  d. Culture the plates at 20°C for 3 days.
  e. After the incubation period, pick animals that survived hygromycin treatment and do not express the mCherry markers to new NGM plates.
    **CRITICAL:** Hygromycin resistant animals should appear healthy and move normally. Many hygromycin sensitive animals will survive but become sterile or remain as larvae or dauer without expressing mCherry – avoid picking these! The presence of embryos in the adults is a good sign that the animal is hygromycin resistant. Also, true hygromycin resistant animals will often be young (L3-L4) at this stage as they can arise in the F2 progeny of F1 treated animals. Refer to troubleshooting, problem 2 for advice.
  f. After ∼2 days, when hygromycin resistant animals have produced progeny, genotype the animals using PCR using primers according Table 1. Ensure the line is homozygous.

**Table 1:** CasSCI insertion genotyping primers

Insertion site	Genotyping primer set	Sequence 5′ to 3′	Annealing location
Chr I (*ttTi4348*)	YJ10507	TGTCGACCGCTAGTGTAGCTTAC	Left homology arm of *ttTi4348* (Chr I)
	YJ10508	CGTCTCTCCACGATTTACACACTATTTG	Right homology arm of *ttTi4348* (Chr I)
	YJ10686	TTTTTCAGAAATATATGCCGAGGATGTTC	Promoter of Hyg resistance gene
Chr IV (*cxTi10882*)	YJ10503	GGAACAAAGGAGTTCAGATCCTGTG	Left homology arm of *cxTi10882* (Chr IV)
	YJ10504	GGAAGACCCTTAGTTCCAAACAAGTG	Right homology arm of *cxTi10882* (Chr IV)
	YJ10686	TTTTTCAGAAATATATGCCGAGGATGTTC	Promoter of Hyg resistance gene
  g. Outcross the single-copy insertion transgenic line(s) as desired.

## Key resources table

**Table:** 

REAGENT or RESOURCE	SOURCE	IDENTIFIER
**Antibodies**		

Anti-FLAG M2 magnetic beads	Sigma-Aldrich	Cat#M8823; RRID: AB_2637089
Anti-FLAG from rabbit (1:2000 recommended dilution)	Sigma-Aldrich	Cat#F7425; RRID: AB_439687
ECL Rabbit IgG, HRP-linked whole Ab from donkey (1:5000 recommended dilution)	Amersham	Cat#NA934

**Bacterial and virus strains**		

DH5α competent cells	Invitrogen	Cat#18258012
*Escherichia coli* OP50	Carolina Biological	Cat#155073

**Chemicals, peptides, and recombinant proteins**		

20× TBS Tween-20 (TBST)	Fisher Scientific	Cat#PI28360
50 bp DNA ladder	Invitrogen	Cat#10416014
5× gel loading dye	Bio-Rad	Cat#1610767
Acid-phenol:chloroform, pH 4.5 (with IAA, 125:24:1)	Thermo Fisher	Cat#AM9720
Agar	Sigma-Aldrich	Cat#05038
Standard agarose	Fisher Scientific	Cat#NC0493156
Ampicillin	Sigma-Aldrich	Cat#A9393
Beta2-mercaptoethanol	Sigma-Aldrich	Cat#M6250
CaCl_2_	Sigma-Aldrich	Cat#C4901
Cholesterol	Sigma-Aldrich	Cat#C8667503
Difco™ Skim Milk	VWR	Cat#90002-594
DMSO	Sigma-Aldrich	Cat#D2650
EDTA	Sigma-Aldrich	Cat#E9884
Ethanol	Sigma-Aldrich	Cat#E7023
Glycerol	Sigma-Aldrich	Cat#G5516
HEPES	Sigma-Aldrich	Cat#54457
Hydrochloric acid (HCl)	Sigma-Aldrich	Cat#H1758
K_2_HPO_4_	Fisher Scientific	Cat#447362500
KCl	Sigma-Aldrich	Cat#P3911
KH_2_PO_4_	Sigma-Aldrich	Cat#P0662
Methanol	Sigma-Aldrich	Cat#34860
MgCl_2_	Sigma-Aldrich	Cat#M8266
Na_2_HPO_4_	Sigma-Aldrich	Cat#S9763
Na_2_HPO_4_,7H_2_O	Sigma-Aldrich	Cat#S9390
NH_4_Cl	Sigma-Aldrich	Cat#213330
Peptone	Fisher Scientific	Cat#DF0118-17-0
SDS	Sigma-Aldrich	Cat#L3771
Sodium chloride (NaCl)	Sigma-Aldrich	Cat#S9888
Sodium hydroxide (NaOH)	Sigma-Aldrich	Cat#S5881
Tris hydrochloride (Tris-HCl)	Sigma-Aldrich	Cat#10812846001
Triton X-100	Sigma-Aldrich	Cat#T8787
TRIzol™ Reagent	Thermo Fisher	Cat#15596026
Tryptone	Sigma-Aldrich	Cat#T7293
UltraPure™ Low Melting Point Agarose	Thermo Fisher	Cat#16520050
Urea	Sigma-Aldrich	Cat#U5378
Yeast extract	Sigma-Aldrich	Cat#Y1625
1 M DTT	Thermo Fisher	Cat#P2325
10× Tris/glycine buffer (running buffer)	Bio-Rad	Cat#1610734
4× Laemmli sample buffer	Bio-Rad	Cat#1610747

**Critical commercial assays**		

T4 RNA ligase reaction buffer	NEB	Cat#B0216SVIAL
AffinityScript cDNA Synthesis Kit	Agilent	Cat#200436
AMPure XP beads	Beckman	Cat#A63880
Buffer RLT	Qiagen	Cat#79216
Dynabeads™ MyOne™ Silane	Thermo Fisher	Cat#37002D
ExoSAP-IT PCR Product Cleanup Reagent	Thermo Fisher	Cat#75001.1.EA
FastAP thermosensitive alkaline phosphatase (1 U/μL)	Thermo Fisher	Cat#EF0654
Gibson Assembly® Master Mix	NEB	Cat#E2611S
Hygromycin B (50 mg/mL)	Thermo Fisher	Cat#10687010
iQ™ SYBR® Green Supermix	Bio-Rad	Cat#1708880
MinElute Gel Extraction Kit	Qiagen	Cat#28604
MinElute PCR Purification Kit	Qiagen	Cat#28004
Murine RNase inhibitor	NEB	Cat#M0314S
Phusion™ Plus DNA Polymerase	Thermo Fisher	Cat#F630S
Pierce™ 10× Western Blot Transfer Buffer	Pierce	Cat#35045
Precision Plus Protein Dual Color Standards	Bio-Rad	Cat#1610374
Proteinase K Solution (20 mg/mL), RNA grade	Thermo Fisher	Cat#25530049
RNA Clean & Concentrator-5	Zymo	Cat#R1013
RNase I, cloned, 100 U/μL	Invitrogen	Cat#AM2294
RNasin™ Plus RNase Inhibitor (RNasin) Sodium chloride (NaCl)	Fisher Scientific Sigma-Aldrich	Cat#PRN2615 S9888
SpeI-HF®	NEB	Cat#R3133S
SphI-HF®	NEB	Cat#R3182S
SuperSignal™ West Pico PLUS Chemiluminescent Substrate	Thermo Fisher	Cat#34579
T4 polynucleotide kinase	NEB	Cat#M0201S
T4 polynucleotide kinase (10 U/μL)	Thermo Fisher	Cat#EK0031
T4 RNA ligase 1, high concentration (supplied with T4 RNA ligase reaction buffer, ATP, and PEG 8000)	NEB	Cat#M0437M
TURBO™ DNase (2 U/μL)	Thermo Fisher	Cat#AM2238

**Deposited data**		

*C. elegans* chromosome sizes	N/A	https://s3-us-west-1.amazonaws.com/genome-references/ce10.chrom.sizes
*C. elegans* reference genome sequence (ce10 STAR index)	N/A	https://s3-us-west-1.amazonaws.com/genome-references/ce10_star_sjdb.tar.gz
Repetitive element STAR index	N/A	https://s3-us-west-1.amazonaws.com/genome-references/STAR_fixed.tar.gz

**Experimental models: Organisms/strains**		

*Caenorhabditis elegans* strain N2 wild type hermaphrodite (mixed developmental stages)	Caenorhabditis Genetics Center	RRID: CGC_N2

**Oligonucleotides**		

Primer annealing to left homology arm of cxTi10882 (YJ10503): GG AACAAAGGAGTTCAGATCCTGTG	This paper	N/A
Primer annealing to right homology arm of cxTi10882 (YJ10504): GGA AGACCCTTAGTTCCAAACAAGTG	This paper	N/A
Primer annealing to left homology arm of ttTi4348 (YJ10507): TGTCG ACCGCTAGTGTAGCTTAC	This paper	N/A
Primer annealing to right homology arm of ttTi4348 (YJ10508): CGTC TCTCCACGATTTACACACTATTTG	This paper	N/A
Primer annealing to promoter of Hyg resistance gene (YJ10686): TTTTTC AGAAATATATGCCGAGGATGTTC	This paper	N/A
InvRiL19 (standard desalted purity; dilute to 40 μM working): /5Phos/rArGrArUrCrGrGrArArGrArGrCrAr CrArCrGrUrC/3SpC3/	Van Nostrand et al.^2^	N/A
InvRand3Tr3 (standard desalted purity; dilute to 80 μM working): /5Phos/NNNNNNNNNNAGATC GGAAGAGCGTCGTGT/ 3SpC3/	Van Nostrand et al.^2^	N/A
InvAR17 (standard desalted purity; dilute to 20 μM working): CAGA CGTGTGCTCTTCCGA	Van Nostrand et al.^2^	N/A
D5×_qPCR (standard desalted purity; dilute to 10 μM working): AATGATACG GCGACCACCGAGATCTACACTATAGC CTACACTCTTTCCCT ACACGACGCTC TTCCGATCT	Van Nostrand et al.^2^	N/A
D7×_qPCR (standard desalted purity; dilute to 10 μM working): CAAGCAGA AGACGGCATACGAGATCGAGTAAT GTGACTGGAGTTCAGA CGTGTGCT CTTCCGATC	Van Nostrand et al.^2^	N/A
D50×_forward (order with desired [i5] index sequence; PAGE purified; dilute to 20 μM working): AATGATA CGGCGACCACCGAGATCTACAC-[i5]-ACACTCTTTCCCTACACGACG CTCTTCCGATCT	Van Nostrand et al.^2^	N/A
D70×_reverse (order with desired [i7] index sequence; PAGE purified; dilute to 20 μM working): CAAGCAGAAG ACGGCATACGAGAT-[i7]-GTGACT GGAGTTCAGACGTGTGCTCTTCCGATC	Van Nostrand et al.^2^	N/A

**Recombinant DNA**		

pCZGY2727 (CasSCI vector Chr I)	Blazie et al.^1^	Deposited to AddGene
pCZGY2729 (CasSCI vector Chr IV)	Andrusiak et al.^8^	RRID: Addgene_135096
pCZGY2748 (sgRNA vector Chr I)	Blazie et al.^1^	Deposited to AddGene
pCZGY2750 (sgRNA vector Chr IV)	Andrusiak et al.^8^	RRID: Addgene_135094

**Software and algorithms**		

CLIPper - CLIP peak enrichment recognition software	Van Nostrand et al.^2^	https://github.com/YeoLab/CLIPper

**Other**		

Immuno-Blot PVDF membrane	Bio-Rad	Cat#1620177
0.2 mL PCR Tubes	Thermo Scientific	Cat#AB-1182
1.5 mL Eppendorf tubes	Denville Scientific	Cat#C2170
15 mL conical tubes	Biopioneer	Cat#CNT-15
Large150 mm Petri dishes (for large NGM plates)	Tritech	Cat#T3325
2 mL Eppendorf tubes	USA Scientific	Cat#1620-2799
4200 TapeStation System	Agilent	Cat#G2991BA
5 mL Eppendorf tubes	Fisher Scientific	Cat#14-282-300
60 mm Petri dishes (for small NGM plates)	Tritech	Cat#T3308
96-well plate seals	Bio-Rad	Cat#MSB1001
96-Well PCR Plates, low profile, unskirted, clear	Bio-Rad	Cat#MLL9601
Dissecting scope	Zeiss	Stemi 2000
DynaMag™-2 Magnet (magnetic rack)	Thermo Fisher	Cat#12321D
Eppendorf™ Thermomixer™ R	Fisher Scientific	Cat#05-400-205
Fluorescence dissecting microscope	Leica	M200
GelDoc XR + UV imager	Bio-Rad	Cat#1708195
Glass pipettes (5 3/4 ″)	Fisher Scientific	Cat#13-678-20B
HiSeq 4000	Illumina	N/A
LabQuake Rotator	Barnstead/Thermolyne	Model 415110
Large centrifuge	Beckman	Allegra X15R
Mini gel electrophoresis system	Fisher Scientific	Cat#09-528-110B
Mini-PROTEAN® Tetra Vertical Electrophoresis Cell for Mini Precast Gels (for SDS PAGE)	Bio-Rad	Cat#1658004
Nitrocellulose membrane (0.45 μM)	Bio-Rad	Cat#1620115
Phase Lock Gel Heavy	VWR	Cat#10847-802
Plastic wrap (Reynolds Food Service Film)	Uline	Cat#S-20200
qPCR Instrument	Bio-Rad	CFX96
Sonicator	QSonica	XL-2000
Spectrolinker crosslinker	Spectronics	XL-1000
Stanley razor blades	Staples	Cat#506923
Tabletop centrifuge	Eppendorf	Model 5424
TGX™ Precast Protein Gels	Bio-Rad	Cat#4569033
Thermal cycler	Bio-Rad	Model T100
Vortex Genie 2 (Votex)	Scientific Industries	SI-0236
Western blot electrophoretic transfer cell (for Western blot transfer; includes sponges and cold pack)	Bio-Rad	Cat#1703930
Whatman paper for blotting	Bio-Rad	Cat#1703965

## Materials and equipment

**Table:** M9 salt buffer

Reagent	Final concentration	Amount
KH_2_PO_4_	20 mM	1.5 g
Na_2_HPO_4_,7H_2_O	20 mM	2.9 g
NaCl	8 mM	0.25 g
NH_4_Cl	20 mM	0.5 g
ddH_2_O	N/A	to 500 mL
**Total**	**N/A**	**50 mL**

Autoclave; store at room temperature; shelf life: > 1 year.

**Table:** Potassium phosphate Buffer (for NGM media)

Reagent	Final concentration	Amount
KH_2_PO_4_ (1 M)	N/A	868 mL
K_2_HPO_4_ (1 M)	N/A	132 mL
**Total**	**N/A**	**1 L**

Filter-sterilize. Store at 4°C for > 1 year.

**Table:** NGM media (agar)

Reagent	Final concentration	Amount
NaCl	N/A	3 g
Agar	N/A	17 g
Peptone	N/A	2.5 g
ddH_2_O	N/A	975 mL
Autoclave to sterilize, allow to cool to 55°C, then add:		
CaCl_2_	1 mM	to 974 mL
Cholesterol (5 mg/mL in 95% EtOH)	N/A	1 mL
Potassium phosphate buffer (1 M)	N/A	25 mL
**Total**	**N/A**	**1 L**

Pour approximately 10 mL per each 10 mm NGM plate and allow agar to solidify before seeding with OP50. Store unseeded plates at 4°C for up to 2 months.

**Table:** LB media with ampacillin (for agar plates)

Reagent	Final concentration	Amount
Tryptone	N/A	10 g
Yeast Extract	N/A	5 g
Agar	N/A	17 g
NaCl	N/A	10 g
ddH_2_O	N/A	to 1 L
Autoclave to sterilize, allow to cool to <55°C, then add:		
Ampicillin (100 mg/mL)	N/A	0.5 mL
**Total**	**N/A**	**1 L**

Store plates at 4°C for up to 3 months.

**Table:** 1× PBS

Reagent	Final concentration	Amount
NaCl	N/A	8 g
KCl	N/A	0.2 g
Na_2_HPO_4_	N/A	1.44 g
KH_2_PO_4_	N/A	0.24 g
ddH_2_O	N/A	to 1 L
**Total**	**N/A**	**1 L**

Autoclave; store at room temperature for over a 1 year.

**Table:** 1× TBS

Reagent	Final concentration	Amount
Tris (1 M, pH 7.5)	50 mM	2.5 mL
NaCl (2 M)	150 mM	3.75 mL
ddH_2_O	N/A	43.75 mL
**Total**	**N/A**	**50 mL**

Store at 4°C for > 1 year.

**Table:** *C. elegans* Lysis Buffer

Reagent	Final concentration	Amount
NaCl (2 M)	150 mM	0.75 mL
HEPES (1 M, pH 7.5)	25 mM	0.25 mL
DTT (1 M)	2 mM	20 μL
Glycerol (30%)	10%	3.33 mL
RNasin	N/A	6.25 μL
Triton X-100 (10%)	4%	4 mL
Nuclease-free H_2_0	N/A	1.5 mL
**Total**	**N/A**	**10 mL**

Add 1 protease inhibitor tablet and incubate at 4°C on rotating wheel for 30 min to dissolve tablet. Prepare fresh each time. May store at 4°C for 3 days.

**Table:** IP Wash Buffer

Reagent	Final concentration	Amount
NaCl (2 M)	N/A	0.75 mL
HEPES (1 M, pH 7.5)	25 mM	0.25 mL
Glycerol (30%)	10%	3.33 mL
Triton X-100 (10%)	4%	4 mL
Nuclease-free H_2_0	N/A	4.67 mL
**Total**	**N/A**	**10 mL**

Add 1 protease inhibitor tablet and incubate at 4°C on rotating wheel for 30 min to dissolve tablet. Prepare fresh each time. May store at 4°C for 3 days.

**Table:** 1× TAP Buffer

Reagent	Final concentration	Amount
Tris (1 M, pH 7.5)	10 mM	0.5 mL
MgCl2 (2 M)	5 mM	125 μL
KCl (1 M)	100 mM	5 mL
Triton X-100 (10%)	0.02%	100 μL
Nuclease-free H_2_0	**N/A**	44.275 mL
**Total**	**N/A**	**50 mL**

Store at 4°C for up to 2 months.

**Table:** 10× Ligase Buffer (no DTT)

Reagent	Final concentration	Amount
Tris-HCl (1 M, pH 7.5)	500 mM	7.5 mL
MgCl_2_ (2 M)	100 mM	0.75 mL
Nuclease-free H_2_0	N/A	6.75 mL
**Total**	**N/A**	**15 mL**

Store at 4°C for up to 2 months.

***Note:*** the 1× Ligase Buffer (no DTT) in our protocol can be prepared by diluting this 10× Ligase Buffer (no DTT) in nuclease free H_2_0.

**Table:** 5× PNK Buffer, pH 6.5

Reagent	Final concentration	Amount
Tris-HCl (1 M, pH 6.5)	350 mM	17.5 mL
MgCl_2_ (2 M)	50 mM	1.25 mL
Nuclease-free H_2_0	**N/A**	31.25 mL
**Total**	**N/A**	**50 mL**

Store at 4°C for up to 2 months.

**Table:** Proteinase K (PK) Buffer

Reagent	Final concentration	Amount
Tris-HCl (1 M, pH 7.4)	100 mM	5 mL
NaCl (2 M)	50 mM	1.25 mL
EDTA (1 M)	10 mM	0.5 mL
SDS (10% in Nuclease-free H_2_0)	0.2%	1 mL
Nuclease-free H_2_0	**N/A**	42.25 mL
**Total**	**N/A**	**50 mL**

Store at 4°C for up to 2 months.

**Table:** 1× Western Transfer Buffer

Reagent	Final concentration	Amount
Pierce™ 10× Western Blot Transfer Buffer	1×	100 mL
100% Methanol	20%	200 mL
Nuclease-free H_2_0	**N/A**	700 mL
**Total**	**N/A**	**1000 mL**

Store at 4°C for up to 2 months.

## Step-by-step method details

### Culturing, crosslinking, and lysis of transgenic *C. elegans*

**Timing: 3–4 days**

**Figure 2:**
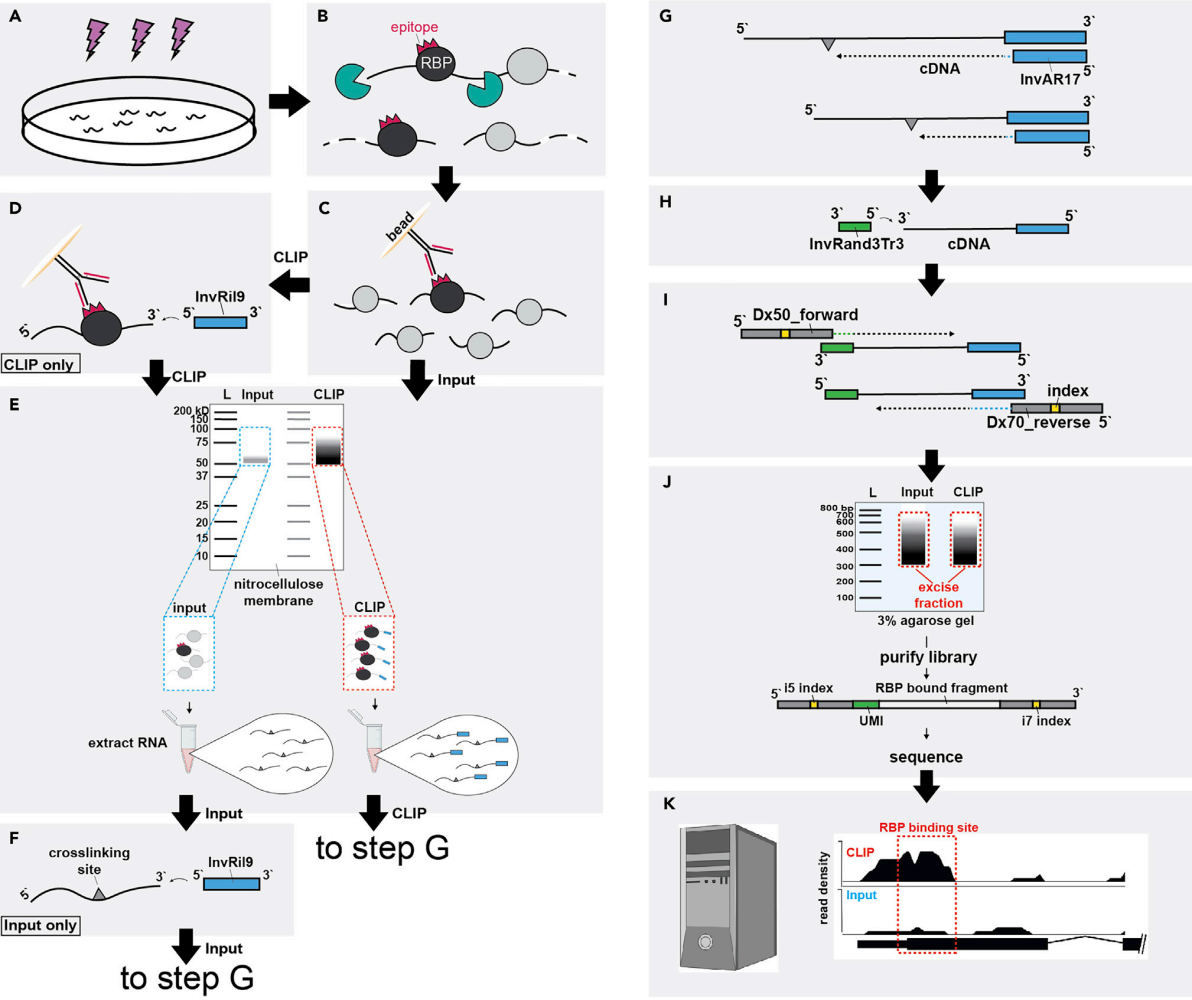
Cell-specific seCLIP protocol flowchart (A) Culture, UV crosslinking, and lysis of transgenic *C. elegans*. (B) RNase treatment of *C. elegans* lysates (RNase: green, target RBP: dark gray, off-target RBPs: light gray). (C) Immunoprecipitation of RBP-RNA complexes. (D) On-bead RNA dephosphorylation and 3′ adapter ligation (CLIP samples ONLY). (E) Western blotting and RNA extraction (CLIP and Input). (F) Input RNA dephosphorylation and 3′ adapter ligation (Input samples ONLY). (G) Generation and clean-up of cDNA (CLIP and Input). (H) 3′ adapter ligation to cDNA (CLIP and Input). (I) PCR amplify and clean-up cDNA library (CLIP and Input). (J) Size select, quantify, and sequence cDNA library (CLIP and Input). UMI: unique molecular identifier used to bioinformatically detect PCR duplicates. (K) Data analysis.

This section describes steps for culturing and harvesting transgenic *C. elegans*, subjecting them to crosslinking to covalently fix the RBP to RNA, and lysing the animals to release cell contents for subsequent RNase treatment (Figure 2A). The number of animals to culture for each seCLIP experiment will depend on several factors, including the RBP transgene expression level, the number of cells expressing the transgene, and the efficiency of immunoprecipitation of the target RBP. As a guideline, we obtained sufficient yields to build high-quality seCLIP libraries with ∼200 μL pelleted *C. elegans* expressing 3×FLAG::EIF-3.G transgene in the cholinergic motor neurons (driven by *Punc-17B*).^1^ Users should adjust the volume of animals depending on the transgene expression variables.

1. Culture each *C. elegans* strain on 12 large NGM plates (150 mm) seeded with OP50 bacteria at the required temperature for 3–4 days.
  a. Pick ∼10 adult hermaphrodites each onto 4 small seeded nematode growth media (NGM) plates (10 cm) and culture 20°C for ∼3 days.
  b. Slice the small plates into quarters and transfer these NGM chunks to 12 large 30 cm NGM plates.
  c. Plates are ready to harvest when they become confluent with *C. elegans*, nearly exhausting the bacterial lawn (typically ∼3–4 days at 20°C).

**CRITICAL:** Before harvesting, briefly examine the animals under a low power dissection microscope to ensure they appear healthy. It is not recommended to harvest *C. elegans* from plates after which the bacterial lawn is exhausted, as many of the animals will begin to experience starvation that could influence RBP activity.

***Note:*** If desired, it is also possible to culture worms from specific larval stages using a modified protocol (for example^12^).

2. Harvest *C. elegans*:
  a. Pour a volume of M9 media enough to cover the surface of the large NGM plate and swirl the solution, allowing the animals to lift off the NGM surface.
  b. Transfer M9 with animals into 15 mL conical tubes.
3. Pellet the animals by centrifugation:
  a. Centrifuge animals at 523 × *g* for 2 min.
  b. Discard the M9 suspension without disturbing the animal pellet.
  c. Resuspend the animals in M9 to 15 mL.
  d. Repeat steps 2 and 3 as necessary until *C. elegans* are harvested from all 12 NGM plates.

***Note:*** Washing animals confluent on 12 large NGM plates typically yields ∼200 μL of pelleted animals.

4. Remove gut bacteria from pelleted animals:
  a. Transfer the animal pellet into a 5 mL Eppendorf tube.
  b. Resuspend the pellet in 5 mL of M9 media.
  c. Attach the 5 mL tube with harvested animals onto a rotator and rotate end to end for 10 min.
5. Wash the animal pellet:
  a. Centrifuge at 523 × *g* for 2 min to pellet animals.
  b. Remove the M9 supernatant and add M9 media to 5 mL.
  c. Repeat steps a and b one time.
6. Prepare animals for crosslinking:
  a. Using a glass pipette, transfer animals onto two large (150 mm) unseeded NGM plates.
  b. Allow the liquid to evaporate and the animals to spread in an even lawn across the plate surface (∼5–10 min).

***Note:*** If needed, animals may be distributed onto more than two large unseeded NGM plates.

7. Pre-cool a small tabletop centrifuge (see key resources table) to 4°C.
8. Insert the animals into the Spectrolinker XL-1000 UV irradiator and initialize crosslinking using an energy setting of 3 kJ/m^2^.

**CRITICAL:** The success of crosslinking can be superficially judged by viewing the *C. elegans* plates under a dissection microscope. Crosslinked animals will be largely immobilized as though they are frozen in place.

9. Wash the crosslinked animals from the large NGM plates:
  a. Pour a volume of M9 media enough to cover the surface of the large NGM plate and swirl the solution, allowing the animals to lift off the NGM surface.
  b. Transfer M9 with animals into 15 mL conical tubes.
  c. Centrifuge animals at 523 × *g* for 2 min.
  d. Discard the M9 suspension without disturbing the animal pellet.
  e. Resuspend the animals in M9 to 15 mL.
  f. Repeat this step as necessary until all *C. elegans* are harvested.
10. Transfer animals:
  a. Remove the M9 supernatant, leaving approximately 5 mL *C. elegans* suspended in M9 media.
  b. Transfer the 5 mL pellet and M9 into a new 5 mL Eppendorf tube using a glass pipette.

***Note:*** The extra volume of M9 merely serves to facilitate transfer of the animals.

11. Pellet animals:
  a. Centrifuge the 5 mL Eppendorf tube with animals at 523 × *g* for 2 min.
  b. Remove as much of the M9 supernatant as possible without disrupting the animal pellet.

**Pause point:** Worm pellets may be flash frozen in liquid nitrogen and stored at −80°C for up to 2 months.

12. Resuspend the animals in 4 mL *C. elegans* lysis buffer and immediately transfer the tube to ice.
13. Sonicate the crosslinked *C. elegans* suspension with seven pulses (10 s each, power setting 11) with 50 s rest on ice in between pulses.

**CRITICAL:** Ensure the samples remain chilled on ice during the entire sonication procedure to avoid overheating the proteins.

14. Clarify lysates:
  a. Clear the lysates by spinning at 5,242 × *g* for 5 min in the small tabletop centrifuge pre- cooled to 4°C.
  b. Carefully move the cleared lysate (4 mL), splitting the volume into four 2 mL Eppendorf tubes (1 mL lysate each).
  c. Discard the insoluble pellet.

***Note:*** Splitting the lysate volume into four tubes serves to promote better mixing during the following RNase treatment steps.

**Pause point:** Lysates may be flash frozen in liquid nitrogen and stored at −80°C for up to 1 week, if desired. Samples may be thawed exactly once when ready to proceed. Avoid multiple freeze thaws.

### RNase treatment of *C. elegans* lysates

**Timing: 25 min**

In this step, clarified lysates are treated with RNase to fragment the total RNA in order to generate RBP associated RNA fragments that will be immunoprecipitated in subsequent steps (Figure 2B).

**CRITICAL:** In all proceeding steps, 'gently flick to mix' refers to manually flicking the reaction tube and firmly tapping the tube on the table surface to gather the liquid to the tube bottom. Keep in mind that excessive force (such as vortexing) can damage RNA and enzymes.

15. Add 2 μL Turbo DNase to each 1 mL lysate sample, gently flick to mix, and return to ice.
16. Dilute RNase I 1:25 in 1× ice-cold PBS.
17. Add 10 μL of the diluted RNase I to each lysate and gently flick to mix.
18. Incubate the lysates in Thermomixer at 37°C for 5 min.
19. Immediately transfer the samples to ice, then add 11 μL Murine RNase Inhibitor and pipette to mix.
20. Centrifuge at 15,000 × g at 4°C for 15 min.
21. Combine the four 1 mL RNase treated lysates reactions into two tubes (2 mL each), which will here forward serve as biological/technical replicates.

Immediately proceed to step 22.

### Immunoprecipitation of RBP-RNA complexes

**Timing: ∼16 h**

In seCLIP, RBP binding sites are identified based on the enrichment of read clusters mapped from the immunoprecipitated RBP (referred to as CLIP) versus an input control (designated as Input). The following steps will describe cDNA library preparation from both CLIP and Input (Figure 2C). We recommend generating and sequencing at least two biological replicate seCLIP libraries for all samples and controls.

22. Aliquot 125 μL anti-FLAG magnetic beads into a 2 mL Eppendorf tube.

***Note:*** The anti-FLAG beads are too viscous to aspirate with most pipette tips. We recommend pipetting the bead suspension with a blunted P200 pipette tip cut with a razor.

23. Wash the anti-FLAG beads (one tube per replicate):
  a. Resuspend the anti-FLAG beads in 500 μL 1× TBS.
  b. Gently vortex the beads.
  c. Magnetically separate the beads (using the magnetic rack; approximately 10 s).
  d. Remove the supernatant.
  e. Repeat steps a through d one time.
24. Add 2 mL of the RNase treated lysate to the beads and incubate at 4°C overnight (∼16 h) on a rotator (total volume ∼2.125 mL).

**Pause point:** Samples are incubated overnight (∼16 h).

25. Remove Input Samples:
  a. Take 20 μL of the beads and lysate sample mixture to a new tube and store at 4°C for the Preparative gel.
  b. Take 20 μL of the beads and lysate sample mixture to a new tube and store at 4°C for the Imaging gel.

**CRITICAL:** These will be used for the western blotting step described in step 44. Keep input samples at −80°C if storing them >1 day.

26. Wash beads:
  a. Magnetically separate beads and remove the supernatant.
  b. Wash beads (thoroughly resuspend them) with 900 μL 1×TBS.
  c. Repeat steps a and b one time.
  d. Magnetically separate beads and remove the supernatant.
  e. Wash beads (thoroughly resuspend them) with 500 μL 1×TBS.
  f. Repeat steps d and e one time.

Immediately proceed to step 27.

### On-bead RNA dephosphorylation and 3′ adapter ligation

**Timing: ∼2 h**

This step will dephosphorylate the immunoprecipitated RNA fragments (CLIP samples only) and ligate the 3′ end with the InvRil19 adapter, which will later be used as a priming site for RT-PCR (Figure 2D). Note that all steps are performed on the beads used for IP.

27. Acclimate beads in 1×TAP buffer:
  a. Remove the 500 μL 1×TBS.
  b. Thoroughly resuspend beads in 500 μL 1×TAP buffer.
  c. Magnetically separate beads and remove the supernatent.
  d. Repeat steps b and c one more time.
28. On ice, prepare the TAP master mix (100 μL per sample):
  a. Add 79 μL H_2_O, 10 μL 10× FastAP Buffer (Thermo Fisher, EF0654), 2 μL Murine RNase Inhibitor, 1 μL Turbo DNase, and 8 μL TAP enzyme to a 1.5 mL eppendorf tube.
  b. Gently flick tube to mix.
29. Add the TAP master mix:
  a. Remove the 500 μL 1×TAP buffer from the last sample wash in step 27.
  b. Add 100 μL of the TAP master mix to each sample.
  c. Incubate in Thermomixer at 1200 rpm, 37°C for 15 min.
30. Prepare the PNK master mix on ice (300 μL per sample): Add 224 μL H_2_O, 60 μL 5×PNK buffer (pH 6.5), 3 μL DTT (0.1 M), 5 μL Murine RNase Inhibitor, 1 μL Turbo DNase, and 7 μL T4 PNK enzyme to a 1.5 mL eppendorf tube.
31. Add 300 μL of the PNK master mix to each sample and incubate in Thermomixer at 1200 rpm, 37°C for 20 min.
32. Magnetically separate the beads and remove the supernatant.
33. Wash once with ice-cold 1×TBS and remove the supernatant.
34. Repeat the wash with 1×TBS five times.
35. Acclimate beads in 1× Ligase Buffer (no DTT):
  a. Resuspend the beads in 500 μL 1× TBS and then add 300 μL 1× Ligase buffer (no DTT).
  b. Gently flick to mix the suspension.
  c. Magnetically separate beads and remove the supernatant.
36. Wash the beads with 1× Ligase buffer (no DTT):
  a. Resuspend beads in 300 μL 1× Ligase buffer (no DTT).
  b. Magnetically separate beads and carefully remove all remaining supernatant.
  c. Repeat steps a and b one more time.
37. Prepare the RNA Adapter Ligation Master Mix on ice (25 μL per sample):
  a. Add 9 μL H_2_O, 3 μL 10× Ligase buffer (no DTT), 0.3 μL ATP (0.1 M), 0.8 μL DMSO (100%), 9 μL PEG 8000 (50%), 0.4 μL Murine RNase Inhibitor, and 2.5 μL High concentration T4 RNA Ligase to a 1.5 mL eppendorf tube.
  b. Gently mix by pipetting.
38. Add 25 μL of the RNA Adapter Ligation Master Mix to each sample.
39. Add 2.5 μL InvRiL19 (40 μM) RNA adapter to each sample.
40. Incubate the samples at room temperature (20°C–23°C) for 75 min and gently flick to mix the reaction every 10 min.

Immediately proceed to step 41.

### Western blotting and RNA extraction

**Timing: ∼22 h**

Western blotting is used to make the target RBP-RNA complex visible so that the RNA can be isolated for cDNA preparation. The seCLIP strategy uses two PAGE gels: the imaging gel is used for western blot detection of the protein to verify the target RBP was successfully immunoprecipitated, and the preparative gel is used for isolation of the RBP-RNA complex. The Input and CLIP samples will be run on both imaging and preparative gels. The RBP-RNA complexes from Input and CLIP will subsequently be isolated from the preparative membrane, the proteins are removed with Proteinase K, and the RNA purified in preparation for subsequent cDNA library generation (Figure 2E).

41. Terminate the ligation reaction:
  a. Magnetically separate beads and entirely remove the supernatant.
  b. Resuspend the beads in 100 μL 1× TBS and pipette to mix.
42. Move 20 μL of each sample to a new 1.5 mL eppendorf tube for the Imaging gel.
43. After magnetically separating the remaining beads and removing supernatant, resuspend the beads in 20 μL cold IP wash buffer.

***Note:*** These samples will be loaded on the Preparative gel.

44. Thaw on ice the imaging and preparative input samples (if stored at −80°C).
45. Prepare NuPAGE mix (per sample): 0.75 μL β-mercaptoethanol + 6.75 μL Laemmli Buffer (4×).
46. To each Input and CLIP sample (20 μL), add: 7.5 μL NuPAGE mix + 3 μL DTT (1 M).
47. Denature all samples:
  a. Incubate all samples for 10 min in a Thermomixer (1,200 rpm at 70°C).
  b. Cool the samples on ice for 1 min before loading on the TGX™ Precast Protein gel.
48. While denaturing samples:
  a. Thaw the protein ladder (we prefer the Precision Plus Dual Color Standard from BioRad).
  b. Prepare a diluted ladder mix for each sample: 2 μL ladder + 2 μL 4× NuPAGE buffer + 6 μL 1× TBS.
49. Load the preparative gel:
  a. Load 13 μL protein ladder (un-diluted) in lane 1.
  b. Load the entire 30 μL volume of preparative samples (both CLIP and Input) in all other lanes with 10 μL diluted ladder mix loaded in between samples.

***Note:*** It is helpful to load the input and CLIP for each sample side by side (with diluted ladder mix in between). The alternating diluted ladder helps to space apart samples in the preparative gel to prevent cross contaminating sampes when protein-RNA complexes are isolated in step 65.

50. Load the imaging gel:
  a. Load 13 μL protein ladder (un-diluted) in the first lane.
  b. Load 15 μL of each imaging gel sample (both CLIP and Input) and save the remaining volume at −80°C as a back-up.

***Note:*** it is not necessary to load diluted ladder between samples in the imaging gel, as the protein-RNA complex will not be excised from the imaging gel.

51. Run the preparative and imaging gels:
  a. Run both gels in 1× Running Buffer at 80 volts for 20 min.
  b. After 20 min, increase voltage to 100 volts and run for 35 additional minutes (or until the dye front is near the bottom of the gel).
52. While the gels are running, prepare 1× western blot transfer buffer and pre-cool at 4°C.
53. Prepare a PVDF membrane for the imaging gel:
  a. Incubate PVDF membrane in 100% methanol for 30 s.
  b. Submerge membrane in ddH_2_O for 30 s.
  c. Leave membrane in transfer buffer (at least 30 s).
54. Prepare a nitrocellulose membrane for the preparative gel by pre-soaking the nitrocellulose membrane in transfer buffer only (Do not submerge nitrocellulose in methanol).
55. For each preparative and imaging gels, assemble the western blot transfer sandwich from anode (black side) to cathode (red) in the following order:
  a. 1 sponge (Biorad, 1703930).
  b. 3 pieces of Whatman paper.
  c. PAGE gel, PVDF (imaging) or Nitrocellulose (preparative) membrane.
  d. 3 pieces of Whatman paper.
  e. 1 sponge.

***Note:*** it is important to keep membrane and sponges wet (avoid letting the membrane dry out) during transfer sandwich assembly.

56. Assemble the transfer cassette:
  a. Carefully roll the western blot transfer sandwich with an empty 15 mL conical tube to remove air bubbles.
  b. Close the transfer cassette and insert into the gel box.
  c. Insert a cold pack (Biorad, 1703930) in the gel box next to the transfer cassette.
  d. Fill the gel box to the top with 1× western blot transfer buffer, ensuring the transfer cassette is completely covered with buffer.
57. Run the western blot transfer at 100 milliampères constant for 90 min or overnight (see note below).

**Pause point:** Alternatively, the transfer may be performed at 30 milliampères overnight (∼16 h) at 4°C.

58. When the transfer is complete, remove the preparative membrane and wash it once with 1× TBS.

**CRITICAL:** Store the membrane in plastic wrap in 4°C until the results of the imaging western blotting are completed.

59. Block the imaging membrane in blocking solution (5% skim milk (w/v) in 1× TBST) at room temperature (20°C–23°C) for 1 h or overnight (∼16 h) at 4°C, with gentle rocking.
60. Add primary antibody to 10 mL total volume of the blocking solution at the desired concentration and incubate overnight (∼16 h) at 4°C with gentle rocking.

***Note:*** We usually start with antibody manufacturer’s recommended concentration and optimize from there. For anti-FLAG (Sigma #F7425), we routinely have success detecting different FLAG-tagged proteins using a 1:2000 dilution in 5% milk in 1× TBST.

61. Wash the membrane 3× with 5% milk in 1× TBST for 5 min each at room temperature (20°C–23°C).
62. Add the secondary antibody at the appropriate concentration (1:5000 for Amersham NA934 anti-Rabbit secondary) in 10 mL total volume 5% milk in 1× TBST and incubate at room temperature (20°C–23°C) for 1 h with gentle rocking.
63. Wash the membrane:
  a. Gently rock membrane with 1× TBST (no milk) for 10 min.
  b. Remove the 1× TBST and add fresh 1× TBST.
  c. Repeat steps a and b three more times.
64. Develop the membrane with ECL using manufacturers recommendations (and image using western blot film of choice.

**Figure 3:**
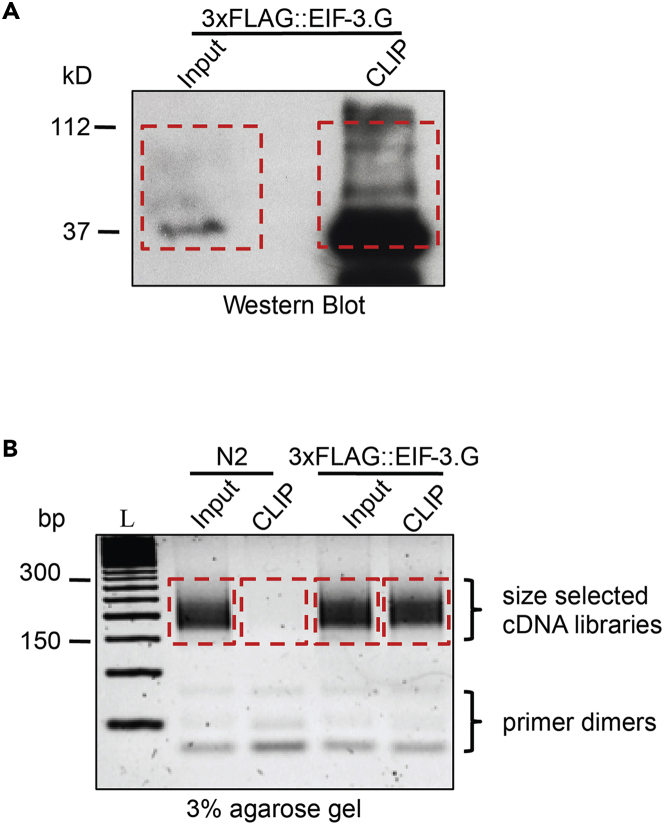
Expected outcomes of western blotting (step 65) and agarose gel cDNA library analysis (step 163) (A) Example of Input and CLIP sample result (3×FLAG::EIF-3.G transgene; (Blazie et al.^1^)) viewed on an imaging western blot. Dotted line shows excised membrane portions. Inputs typically yield less signal than CLIP sample due to Lower tagged-RBP concentration. (B) cDNA library amplification products from N2 (no transgene control) and 3×FLAG::EIF-3.G run on a 3% agarose gel. Dotted line indicates excised fractions. DNA smearing is evident in all lanes except the N2 control CLIP sample, which lacks the tagged-RBP. L = 50 bp DNA ladder.

**CRITICAL:** Take note of the position of the tagged-RBP in the IP samples relative to the protein ladder as this will be used in subsequent steps (Refer to Figure 3A for an example). Refer to troubleshooting, problems 3 and 4 for advice.

65. Excise the RBP-RNA complex:
  a. Place the preparative membrane on a glass surface.
  b. Using the developed imaging gel as a guide, slice the IP sample from the tagged-RBP band including 75 kD above this band (Refer to Figure 3A).
  c. Carefully slice this membrane into ∼1 mm strips, transfer all strips into a single clean 1.5 mL Eppendorf tube and place the tube on ice.
66. Prepare proteinase K (PK) mix on ice (200 μL per sample): 160 μL PK buffer + 40 μL Proteinase K Solution.
67. Prepare Urea/PK buffer:
  a. Dissolve 420 mg Urea in 500 μL PK buffer.
  b. Add PK buffer to a final volume of 1 mL.
68. Submerge the membrane slices in 200 μL of the PK mix and incubate for 5 min in the Thermomixer (1,200 rpm at 37°C).
69. Incubate samples in Urea/PK buffer:
  a. Add 200 μL of the Urea/PK buffer to the samples.
  b. Gently flick tubes to mix.
  c. Incubate the samples for 20 min in the Thermomixer (1,200 rpm at 37°C).
70. Incubate samples with Acid-Phenol:Chloroform reagent:
  a. In a laminar flow cabinet, add 400 μL Acid-Phenol:Chloroform, pH 4.5 to the samples.
  b. Mix samples thoroughly by inverting the tubes.
  c. Incubate samples in the Thermomixer (1,200 rpm) at 37°C for 5 min.
71. Transfer all liquid (leave the slices) to a Heavy Phaselock gel tube and incubate in the Thermomixer at 1,200 rpm 37°C for 5 min.
72. Centrifuge at 13,000 × *g* for 15 min at room temperature (20°C–23°C).
73. Transfer the top aqueous layer (typically ∼400 μL) to a 15 mL conical tube and add 2 volumes of RNA binding buffer (Zymo RNA Clean and Concentrator kit).
74. Add an equal volume (of the aqueous layer, typically ∼400 μL) of 100% ethanol and mix well.
75. Capture RNA in a Zymo-Spin column:
  a. Transfer 750 μL to a Zymo-Spin column.
  b. Centrifuge for 30 s at 13,000 × *g.*
  c. Discard the flowthrough.
  d. Repeat steps a through c until all the sample has spun through the column.
76. Add 400 μL RNA Prep Buffer, centrifuge for 30 s, and discard the flowthrough.
77. Add 700 μL RNA Wash Buffer, centrifuge for 30 s, and discard the flowthrough.
78. Add 400 μL RNA Wash Buffer, centrifuge for 30 s, and discard the flowthrough.
79. Centrifuge the column for 2 min to remove all remaining buffer.
80. Place the column into a new 1.5 mL Eppendorf tube.
81. Elute RNA:
  a. Add 10 μL nuclease-free H_2_O to the column and incubate at room temperature (20°C–23°C) for 1 min.
  b. Centrifuge for 30 s at 13,000 × *g.*

**Pause point:** Samples may be stored at −80°C until ready to proceed.

### Input RNA dephosphorylation and 3′ adapter ligation (input samples ONLY)

**Timing: 3 h (for steps 82 to 109)**

This step dephosphorylates RNA in the Input samples and then ligates the InvRil19 adapter to the 3′ RNA ends (Figure 2F). The InvRil19 adapter will be subsequently used as a priming site for RT-PCR.

82. Prepare the TAP master mix (for each sample): 10 μL H_2_O, 2.5 μL 10× FastAP Buffer (Thermo Fisher, EF0654), 0.5 μL Murine RNase Inhibitor, 2.5 μL TAP enzyme.
83. **To INPUT samples ONLY:** add 15.5 μL TAP master mix.
84. Prepare the PNK master mix (for each sample): 45 μL H_2_O, 20 μL 5× PNK Buffer (pH 6.5), 1 μL DTT (0.1 M), 1 μL DNase, 1 μL Murine RNase Inhibitor, 7 μL T4 PNK enzyme.
85. Add 75 μL of the PNK master mix to each sample, flick tubes to mix, and incubate in Thermomixer at 1,200 rpm, 37°C for 20 min.
86. Prepare MyONE Silane beads: magnetically separate 20 μL MyONE Silane beads per sample and remove the supernatant.
87. Resuspend the beads in 900 μL RLT buffer, separate, and remove the supernatant.
88. Resuspend the beads in 300 μL RLT buffer and transfer the entire volume (300 μL + beads) to the sample tube and mix well by pipetting.
89. Add 10 μL NaCl (5 M).
90. Add 615 μL 100% EtOH.
91. Mix well by pipetting and rotate at room temperature (20°C–23°C) for 15 min.
92. Wash once with 75% EtOH:
  a. Magnetically separate the beads and remove all supernatant.
  b. Add 1 mL 75% EtOH.
  c. Move the resuspended beads to a new tube and allow the beads to sit for 30 s at room temperature (20°C–23°C) in the new tube.
93. Wash two more times with 75% EtOH:
  a. Magnetically separate beads and remove all supernatant.
  b. Resuspend beads in 75% EtOH.
  c. Allow beads to sit for 30 s at room temperature (20°C–23°C).
  d. Repeat steps a through c one more time.
94. Magnetically separate beads, thoroughly remove all supernatant using a fine pipette tip, and air dry the beads for exactly 5 min.
95. Resuspend the dry beads in 5 μL H_2_O and incubate at room temperature (20°C–23°C) for 5 min.
96. Magnetically separate the beads and transfer 5 μL of the eluted RNA (supernatant) for the steps below.

**CRITICAL:** Save the remaining 5 μL in −20°C as backup.

97. Add 1.5 μL 100% DMSO and 0.5 μL InvRiL19 (40 μM) adapter to the 5 μL eluted RNA.
98. Incubate at 65°C for 2 min and immediately place on ice.
99. Prepare ligation master mix (13.5 μL per sample): 2 μL 10× T4 RNA Ligase Reaction Buffer, 0.2 μL ATP (0.1 M), 0.2 μL Murine RNase Inhibitor, 0.3 μL 100% DMSO, 8 μL 50% PEG 8000, 1.3 μL RNA Ligase high concentration, 1.5 μL nuclease free H_2_O.
100. Ligate the adapter:
  a. Add 13.5 μL ligation master mix to each sample.
  b. Gently flick tubes to mix.
  c. Incubate samples at room temperature (20°C–23°C) for 75 min.
  d. Gently mix tubes by flicking every 15 min.
101. For each sample, magnetically separate 20 μL MyONE Silane beads and remove the supernatant.
102. Add beads in RLT to the samples:
  a. Wash the 20 μL MyONE Silane beads once with 900 μL RLT buffer.
  b. Resuspend the beads in 61.6 μL RLT buffer.
  c. Add the beads in RLT to the samples and mix.
103. Wash in 100% EtOH:
  a. Add 61.5 μL 100% EtOH to each sample.
  b. Mix by pipetting and leave the pipette tip in the sample tube.
  c. Incubate for 15 min at room temperature (20°C–23°C), pipette mixing every 5 min.
104. Magnetically separate beads and remove the supernatant.
105. Wash with 75% EtOH:
  a. Add 1 mL 75% EtOH to the beads and pipette to mix.
  b. Move the entire resuspension to a new tube.
  c. Incubate the resuspension for 30 s at room temperature (20°C–23°C).
  d. Magnetically separate beads and remove the supernatant.
106. Repeat the wash with 75% EtOH (step 105) two more times.
107. Dry the beads:
  a. Magnetically separate beads and remove all liquid using a fine tip.
  b. Air dry the beads for 5 min at room temperature (20°C–23°C).
108. Resuspend the beads in 10 μL H_2_O and incubate at room temperature (20°C–23°C) for 5 min.
109. Magnetically separate beads and transfer the 10 μL supernatant to a new tube (this is your RNA).

Immediately proceed to step 110.

### Generation of CLIP and input cDNA

**Timing: 75 min (for steps 110 to 126)**

cDNA will be generated with the InvAR17 primers, which anneals to the InvRil19 adapter ligated to CLIP and Input RNA samples (Figure 2G).

110. For ALL samples (CLIP and Input): mix 10 μL RNA with 0.5 μL InvAR17 (20 μM) primer in a 0.2 mL PCR tube.
111. Incubate the tubes at 65°C for 2 min in a pre-heated thermocycler and then immediately transfer to ice.
112. Prepare reverse transcription master mix (on ice; 10 μL per sample; Reagents are included in the AffinityScript cDNA Synthesis kit):
  a. For each sample, mix 4 μL H_2_O, 2 μL 10× AffinityScript Buffer, 2 μL DTT (0.1 M), 0.8 μL dNTPs (100 mM), 0.3 μL Murine RNase Inhibitor, 0.9 μL AffinityScript Enzyme into a 1.5 mL eppendorf tube.
  b. Mix well by flicking tube.
113. Add 10 μL master mix to each sample, mix well, and incubate at 55°C for 45 min in a pre-heated thermocycler.
114. Incubate samples with ExoSAP-IT:
  a. Add 3.5 μL ExoSAP-IT mix to each sample.
  b. Vortex and briefly spin down.
  c. Incubate samples at 37°C for 15 min in a thermocycler.
115. Add 1 μL EDTA (0.5 M) and pipette mix.
116. Add 3 μL NaOH (1 M) and pipette mix.
117. Incubate samples at 70°C for 12 min in thermocycler.
118. Add 3 μL HCl (1 M) and pipette mix.
119. Magnetically separate 10 μL MyONE Silane beads (from the stock bottle) per sample and remove the supernatant.
120. Add beads in RLT buffer to the samples:
  a. Wash the 10 μL MyONE Silane beads once with 500 μL RLT buffer.
  b. Resuspend the beads in 93 μL RLT buffer.
  c. Add the entire volume of beads in RLT to the sample.
  d. Gently flick to mix the samples.
121. Wash with 100% EtOH:
  a. Add 111.6 μL 100% EtOH and pipette to mix, leaving pipette tip in the tube.
  b. Incubate samples for 5 min at room temperature (20°C–23°C), pipetting mix twice during incubation.
122. Magnetically separate beads and remove the supernatant.
123. Wash with 80% EtOH:
  a. Add 1 mL 80% EtOH, pipette mix, and move the resuspension to a new tube.
  b. Incubate at room temperature (20°C–23°C) for 30 s.
  c. Magnetically separate beads and remove the supernatant.
124. Repeat washes in 1 mL 80% EtOH (step 123) two more times.
125. Magnetically separate samples, remove all liquid with a fine tip, and air dry for 5 min.
126. Resuspend beads in 5 μL Tris-HCl (5 mM, pH 7.5) and incubate for 5 min at room temperature (20°C–23°C).

**CRITICAL:** Do not remove liquid from beads.

Immediately proceed to step 127.

### 3′ linker ligation to cDNA

**Timing: 17 h (for steps 127 to 145)**

This step will ligate the InvRand3Tr3 adapter to the 3′ end of the newly generated cDNA (Figure 2H). InvRand3Tr3 will later serve as an annealing site for the D50×_forward primer used to amplify cDNA libraries for sequencing on the Illumina instrument. InvRand3Tr3 additionally contains a short, randomized sequence (Unique Molecular Identifier) that serves as a barcode to later distinguish cDNA molecules generated from unique RNA fragments from PCR duplicated cDNAs during the computational analysis of sequencing data.

127. Add 0.8 μL InvRand3Tr3 (80μM) adapter to each 5 μL sample.
128. Add 1 μL 100% DMSO.
129. Incubate at 75°C for 2 min, then immediately transfer tube to ice.
130. Prepare ligation master mix on ice (12.8 μL per sample): 2 μL 10× T4 RNA Ligase Reaction Buffer, 0.2 μL ATP (0.1 M), 9 μL 50% PEG 800, 0.5 μL High concentration T4 RNA Ligase, 1.1 μL H_2_O.
131. Add ligation mix to samples:
  a. Gently flick master mix tube to mix.
  b. Tap the tubes on the table surface to gather liquid to the tube bottom.
  c. Add 12.8 μL ligation master mix to each sample.

**CRITICAL:** Pipette the reaction, ensuring the sample is thoroughly mixed with the master mix.

132. Add an additional 1 μL High Concentration T4 RNA Ligase to the sample and pipette to mix.
133. Incubate the reactions at room temperature (20°C–23°C) on a rotator overnight (∼16 h).

**Pause point:** Samples are incubated overnight (∼16 h).

134. Next day: magnetically separate 5 μL MyONE Silane beads per sample and remove the supernatant.
135. Wash the beads once with 500 μL RLT buffer.
136. Resuspend the beads in 60 μL RLT buffer per sample.
137. Transfer 60 μL of beads in RLT buffer to each sample, gently flick to mix, and add 60 μL 100% EtOH.
138. Pipette to mix and incubate at room temperature (20°C–23°C) for 5 min.

**CRITICAL:** Pipette mix the samples twice during the incubation period.

139. Magnetically separate beads and remove the supernatant.
140. Wash the beads in 75% EtOH:
  a. Resuspend the beads thoroughly in 1 μL 75% EtOH.
  b. Move the entire volume to a new 1.5 mL Eppendorf tube.
  c. Incubate at room temperature (20°C–23°C) for 30 s.
141. Magnetically separate beads and remove the supernatant.
142. Repeat the wash with 75% EtOH (steps 140 and 141) two more times.
143. Dry the beads:
  a. Magnetically separate beads and remove all supernatant with a fine tip.
  b. Air dry the beads for 5 min at room temperature (20°C–23°C).

**CRITICAL:** Ensure that all ethanol has evaporated from the beads, as residual ethanol can inhibit downstream reactions.

144. Resuspend the beads in 27 μL Tris-HCl (10 mM, pH 7.5) and incubate at room temperature (20°C–23°C) for 5 min.
145. Magnetically separate beads and transfer 25 μL of the supernatant to a new 1.5 mL Eppendorf tube.

Immediately proceed to step 146.

### Quantify cDNA, PCR amplify and clean-up cDNA library

**Timing: 3 h (for steps 146 to 158)**

qPCR is used to estimate the quantity of template cDNA in order to determine the number of PCR cycles needed for PCR amplification of the cDNA library. Note that it is not necessary to perform the qPCR in replicates. If desired to confirm accuracy of qPCR results, users can perform a duplicate PCR using a 1:100 dilution of the sample cDNA, which should yield a C_T_ ∼3 cycles above the 1:10 cDNA dilution. After qPCR, cDNA libraries are generated using primers containing indexes for Single-end sequencing on the Illumina HiSeq instruments (Figure 2I). The PCR reactions are cleaned-up using magnetic MyOne Silane beads to prepare for subsequent cDNA library size-selection and purification.

146. Prepare a 1:1 mix of D5× and D7× qPCR primers (10 μM each, 0.4 μL per sample).
147. Prepare the qPCR master mix on ice (9 μL per sample): 5 μL qPCR 2× master mix, 3.6 μL H_2_O, 0.4 μL D5×/D7× primer mix.

***Note:*** Although not strictly required, it may also be useful to add a 'no template' control qPCR reaction with 1 μL H_2_O added instead of sample cDNA. The no template control should not yield fluorescent signal if the qPCR reagents are working properly.

148. Dispense master mix (9 μL per sample) into a 96-well PCR plate.
149. Add 1 μL sample cDNA (1:10 diluted in H_2_O) to the reaction mix, seal (BioRad, #MSB1001) the plates and mix gently.
150. Run qPCR according to the instrument instructions.

**CRITICAL:** Record the C_T_ values obtained for each sample. Use this C_T_ to determine the number of PCR cycles needed to amplify the cDNA library (below): Total PCR cycles = C_T_ value (from qPCR) – 3.

151. Prepare PCR master mix on ice (37.5 μL per sample):

**Table:** 

Reagent	Amount
iQ™ SYBR® Green Supermix	25 μL
nuclease free H_2_O	7.5 μL
D50×_forward primer (20 μM)	2.5 μL
D70×_reverse primer (20 μM)	2.5 μL

Gently flick tube to mix.

**CRITICAL:** In D50× and D70× primers, the 'x' represents a unique index that can be used to multiplex samples on the Illumina sequencing platform (see Oligonucleotides Section in key resources table). Therefore, you may desire to prepare each sample with a unique index combination (e.g., sample 1: D501/D702, sample 2: D502/D701, etc.) for subsequent sample pooling before library sequencing.

152. Prepare the qPCR reactions:
  a. Dispense 37.5 μL of the PCR master mix into 0.2 mL PCR Tubes.
  b. Add 12.5 μL of sample cDNA and mix well by flicking tubes.
  c. Spin down the reaction tubes.
153. Perform PCR using the following cycle conditions (cycle # depends on qPCR Ct as determined above):

**Table:** 

Steps	Temperature	Time	Cycles
Initial Denaturation	98°C	30 s	1
Denaturation	98°C	15 s	6 cycles
Annealing	68°C	30 s	
Extension	72°C	40 s	
Denaturation	98°C	15 s	qPCR Ct minus 9 cycles
Annealing and Extension	72°C	60 s	
Final extension	72°C	1 min	1
Hold	4°C	forever

154. Purify DNA:
  a. Add 90 μL AmpureXP beads suspension (do not separate) per 50 μL PCR reaction and mix thoroughly by pipetting.
  b. Incubate at room temperature (20°C–23°C) for 10 min, mixing the sample 3 times during the incubation by pipetting up and down.
155. Wash with 75% EtOH:
  a. Magnetically separate beads.
  b. Wash twice with 75% EtOH.
  c. Remove the supernatant and airdry beads for 5 min.

**CRITICAL:** Ensure that all ethanol has evaporated from the beads, as residual ethanol can inhibit downstream reactions.

156. Resuspend beads in 20 μL H_2_O and incubate at room temperature (20°C–23°C) for 5 min.
157. Magnetically separate beads for an additional 5 min.
158. Transfer 18 μL of supernatant to a new tube.

**Pause point:** Samples may be stored at −20°C for up to two weeks.

### Size select, quantify, and sequence cDNA library

**Timing: 1.5 h (for steps 159 to 166)**

In this step, cDNA libaries will be electrophoretically seperated on a 3% agarose gel, the DNA size-selected (175–300 bp) and gel excised, and purified in preparation for single-end sequencing on the Illumina platform (Figure 2J).

159. Prepare a 3% low-melting temp agarose gel in 1×TBE with EtBr.
160. Add 4.5 μL 5× Gel Loading Dye to each 18 μL sample.
161. Load samples onto the agarose gel:
  a. Carefully load the entire 22.5 μL sample on the gel, leaving one empty lane between samples.
  b. Load 5 μL of 50 bp DNA ladder on both ends of the gel.
162. Run gel at 95 V for 50 min.

***Note:*** this will depend on the size of the gel box. Longer running times result in better resolution but require the user to cut larger agarose slices to purify the library.

163. Briefly image the gel under UV light.

***Note:*** You should see a smear between ∼50 bp to 800 bp in sample lanes (Refer to Figure 3B for an example). See troubleshooting, problem 5 for advice.

**CRITICAL:** Minimize the time gels are exposed to UV as it can damage the cDNA.

164. With a clean razor blade, excise the gel slice from 175–350 bp and place into a 15 mL conical tube.
165. Excise and elute the gel using the Qiagen MiniElute gel extraction kit according to the following:
  a. Weigh the gel slice to determine volumes needed for following steps.
  b. Add 6× volumes of Buffer QG to melt the gel (e.g., for 100 mg gel slice, add 600 μL QG).
  c. Weigh the gel slice.
  d. Melt the gel at room temperature (20°C–23°C; do not heat, do not vortex) and gently shake tube to facilitate gel melting.
  e. Add 1× volume of the original gel of 100% isopropanol and mix well (100 mg gel = 100 μL isopropanol).
  f. Capture DNA in MiniElute column:
    i. Load 750 μL into a MiniElute column.
    ii. Spin at max speed for 1 min.
    iii. Repeat loading and spinning the sample as necessary until the entire sample has spun through the column.
  g. Wash the column once with 500 μL Buffer QG.
  h. Wash the column with Buffer PE:
    i. Add 750 μL Buffer PE.
    ii. Spin at max speed for 1 min.
    iii. Discard the flowthrough and spin column again for 2 min at max speed.
  i. Transfer the column to a fresh 1.5 mL Eppendorf tube and let it air dry for 2 min.
  j. Carefully add 12.5 μL Buffer EB to the center of the column, incubate for 2 min at room temperature (20°C–23°C), and spin at max speed for 1 min.

**Pause point:** Eluted sample libraries may be stored at −80°C for up to one month before quantitation and sequencing.

166. Proceed to quantitate library on the Agilent TapeStation and deep sequence libraries on the Illumina HiSeq instrument according to your Sequencing Core instructions.

### Data analysis

**Timing: 3 h (for steps 167 to 169)**

Illumina HiSeq outputs seCLIP sample sequence data in FASTA format. CLIPper is a dedicated seCLIP analysis software developed and maintained by the Yeo Lab, which will trim adapters, filter ambiguous mapping reads, and remove PCR-duplicate reads from the raw sequences.

167. Save the FASTA file outputs for each sample (Input and CLIP) to a new directory where you will execute CLIPper.
168. CLIPper requires several user input *C. elegans* reference sequence files along with your seCLIP sequence data (save these files in the same directory where you will invoke CLIPper):
  a. *C. elegans* reference genome sequence (ce10 STAR index): https://s3-us-west-1.amazonaws.com/genome-references/ce10_star_sjdb.tar.gz.
  b. *C. elegans* chromosome sizes: https://s3-us-west-1.amazonaws.com/genome-references/ce10.chrom.sizes.
  c. Repetitive element STAR index: https://s3-us-west-1.amazonaws.com/genome-references/STAR_fixed.tar.gz.
169. Download and invoke CLIPper according to the latest documentation: https://github.com/YeoLab/CLIPper.

***Note:*** After trimming adaptors and filtering reads, CLIPper will align the remaining high-quality reads to the *C. elegans* reference genome to generate alignment maps (BAM files). CLIPper will identify RBP binding sites from the sequence maps on the basis of read cluster enrichment in CLIP versus Input samples (Figure 2K) and outputs these clusters in .BED format (see quantification and statistical analysis below).

## Expected outcomes

The success of cell-specific seCLIP in *C. elegans* should be carefully monitored during protocol execution. On the imaging western blot, the immunoprecipitated RBP will generally yield a smear between the expected RBP molecular weight and 75 kD above (Figure 3A). However, RBP signals in the Input samples appear faint because the RBP concentration is much less than in CLIP (Figure 3A). Successful generation of cDNA libraries are indicated by a DNA smear on the agarose gel between ∼150–300 bp and relative absence of DNA in CLIP samples from the ‘no transgene’ control (Figure 3B).

Evidence of seCLIP success is also apparent in CLIPper data output. Users should beware that CLIPper generally filters >50% of the total raw reads because it is extremely selective for high quality, uniquely mapping reads (Van Nostrand et al., 2017). However, each sample (CLIP and Input) should generally yield at least ∼1 M high quality reads after filtering to allow sufficient identification of read clusters representing RBP binding sites. The cell-specificity of RBP-binding sites can be assessed by comparing the overlap between all seCLIP identified RBP target genes with previously published cell-specific transcriptome datasets (e.g., (Blazie et al.^4^), (McCulloch et al., 2020^13^), (Gracida and Calarco^6^)).

## Quantification and statistical analysis

CLIPper identifies RBP binding sites from the alignment maps as statistically enriched read clusters (peaks) in CLIP samples relative to the Input control samples. CLIPper will output a raw list of RBP peaks along with their genomic position interval, enrichment (log foldchange) in CLIP/Input, and statistical confidence (P-value) in BED file format. The parameters in the BED files may be used to custom set statistical thresholds to prioritize RBP binding sites. As described above, it is often useful to perform seCLIP with a control RBP transgene deficient in RNA-binding activity (i.e., mutated RNA-binding domain) and a control strain without the RBP transgene (e.g., N2 strain). The RBP peaks detected from the control datasets can be considered non-specific background and ignored in the sample data set. The remaining peaks can be regarded with higher confidence as true signal.

## Limitations

The sensitivity and specificity of cell-specific seCLIP highly depends on the efficiency of RBP immunoprecipitation. Key factors limiting IP success include the number of cells expressing the RBP, RBP expression level, RBP size and solubility, and the performance of the antibody used for IP. Therefore, we recommend considering which of these factors may limit your application and optimizing IP before implementing seCLIP. Users should also beware that seCLIP will inevitably yield some off-target or artifactual RBP binding sites even when rigorous controls were included. It is therefore prudent to validate the most interesting RBP-binding sites with secondary experimental approaches.

## Troubleshooting

### Problem 1

Poor yields of the CasSCI vector (pCZGY2727 or pCZGY2729) are obtained after miniprep (before you begin, CasSCI transgenesis, step 1).

### Potential solution

We have sometimes observed poor miniprep efficiency of the CasSCI vector (without any DNA inserts of desired transgene) and have identified two solutions. 1) The CasSCI vectors contain a ccdB cassette and DB3.1 bacteria containing these plasmids propagate slowly. Grow the liquid bacteria culture (3 mL in LB with Ampicillin and Chloramphenicol) for 48 h (instead of 16 h) before miniprep. 2) When eluting DNA from the Qiagen miniprep column, add 50 μL of elution buffer preheated to 70°C and let the column sit at room temperature (20°C–23°C) for 2 min before spinning.

### Problem 2

No single-copy insertion transgenic lines are obtained (all transgenic animals contain the extrachromosomal array markers and/or are sensitive to hygromycin) during CasSCI (before you begin, CasSCI transgenesis, step 3e).

### Potential solution

Reduce the concentration of the CasSCI vector (containing the tagged-RBP) in the injection mix. We have obtained lines using between 5–15 ng/μL CasSCI vector, depending on the toxicity associated with RBP overexpression.

Inject into wild type (N2) hermaphrodites as mutant backgrounds could reduce CasSCI efficiency.

Inject at least 50 P0 hermaphrodites as the CasSCI insertion efficiency may be low for some transgenes.

Consider reducing the transgene size, if possible. We have observed reduced CasSCI insertion efficiencies with transgenes >3,500 bp.

### Problem 3

Tagged-RBP is not detected on the imaging western blot (step 64).

### Potential solution

If proteins are not observed on a western blot, we recommend quantifying the protein concentration resulting from *C. elegans* lysis (after step 14) using approaches such as Bradford or BSA assays. We routinely obtain protein concentrations between 20–40 mg/mL from whole *C. elegans* lysates. We have observed that a clarified *C. elegans* lysate (after pelleting the lysis) will be golden brown in color and have used this to superficially judge the quality of lysis before subjecting samples to seCLIP. Lysates with poor protein yields will have a color closely resembling the starting lysis buffer (clear in color; not yellow or brown).

Ensure that protease inhibitors are added to the lysis buffer.

Check a large volume of the input lysate on a western blot to ensure that your RBP is sufficiently expressed and detectable.

Optimize concentrations of the primary and secondary antibody used for western blotting. Also, consider using a more sensitive western blotting detection agent (e.g., Femto ECL) for low abundance proteins.

Include a positive control tagged protein (it doesn’t need to be an RBP) that has worked in previous western blotting applications to verify the IP and western blot reagents are working.

### Problem 4

Additional signals of unexpected size appear on the western blot (step 64).

### Potential solution

If genomic DNA was used to clone the RBP transgene, additional bands on the westen blot might indicate that the protein of interest encodes multiple protein isoforms. Consider performing seCLIP with an RBP trasngene encoded from a cDNA of the desired RBP protein isoform.

Ensure that fresh protease inhibitors were added to they lysis buffer to avoid RBP degradation.

Increase the salt concentration (NaCl) of the IP wash buffer and/or the number of washes after IP.

Optimize the concentration of the primary and/or secondary antibody used for western blotting. Excessive antibody concentration or incubation periods can result in high background.

### Problem 5

No DNA smear is detected in the agarose gel in step 163.

### Potential solution

Ensure that fresh RNAse inhibitors were added at each step where indicated.

Verify that all enzymes used to prepare the cDNA library (i.e., AffinityScript RT polymerase, RNA ligase, etc.) are working. Many commercial manufacturers will include a positive control that can be used to check enzyme performance. We recommend always using fresh enzyme stocks.

Repeat seCLIP with a positive control tagged-RBP (e.g., 3×FLAG::EIF-3.G) to ensure the reagents are working.

## Resource availability

### Lead contact

Further information and requests for resources and reagents should be directed to and will be reasonably fulfilled by the lead contact, Yishi Jin (yijin@ucsd.edu).

### Materials availability

All genetic constructs and *C. elegans* strains are available upon request to the lead contact.

## Data Availability

All datasets generated in our published application of cell-specific seCLIP (Blazie et al.^1^) are available from the Gene Expression Omnibus, accession number GEO: GSE152704.
